# To modify or not to modify—That is still the question for some mRNA applications

**DOI:** 10.1016/j.omtn.2025.102655

**Published:** 2025-08-11

**Authors:** Sofía Soler, Katharina Maser, Thomas Zillinger, Eva Bartok

**Affiliations:** 1 Institute of Experimental Haematology and Transfusion Medicine, University Hospital Bonn, Bonn, Germany; 2 Institute of Clinical Chemistry and Clinical Pharmacology, University Hospital Bonn, Bonn, Germany

## Main text

**Figure 1:**
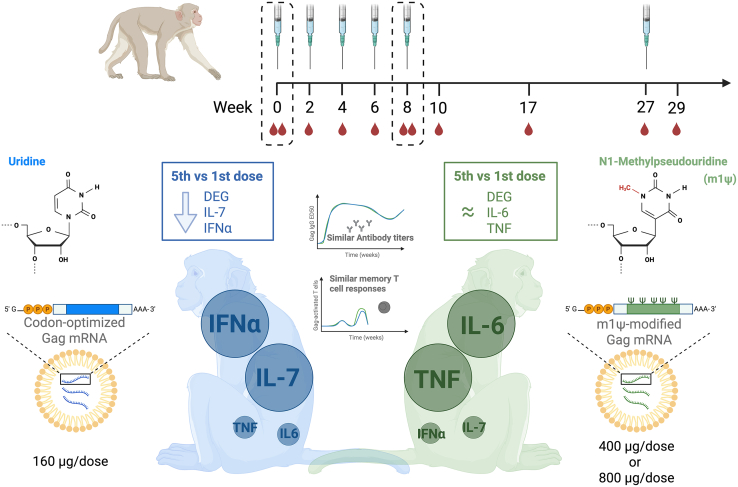
Innate and adaptive immune responses to high-dose, repetitive immunization in NHPs using codon-optimized, unmodified versus m1Ψ-modified HIV Gag mRNA vaccines Upper panel: immunization regimen applied by Engstrand et al. Rhesus macaques were immunized five times at 2-week intervals, and again at week 27, using sequence-optimized, unmodified (160 μg) or m1Ψ-modified (400 or 800 μg) HIV-1 Gag mRNA. Blood was analyzed for cytokine levels, memory T cell populations, and transcriptomic changes at indicated time points (red droplets). Lower panel: comparison of innate and adaptive immune responses to unmodified (left side, blue) and modified (right side, green) mRNA observed by Engstrand et al. mRNA vaccine containing unmodified uridine induced higher levels of IFNα and IL-7, while m1Ψ-modified mRNA stimulated stronger IL-6 and TNF induction. Furthermore, unmodified mRNA induced lower cytokine levels after the fifth dose compared to the first dose (blue box), indicating induction of immune tolerance, while m1Ψ-modified mRNA cytokine responses were similar between first and fifth injection (green box). Despite the different innate responses, antibody titers and memory T cell responses were similar for both mRNA vaccines (center).

In their recent study,^1^ Engstrand and colleagues evaluated the innate and adaptive responses to high-dose unmodified and N1-methylpseudouridine-modified (m1Ψ) mRNA vaccination using a Gag-antigen model in non-human primates (NHPs) (Figure 1). Direct comparison revealed overwhelmingly similar antibody levels over time and comparable, though relatively weak, T cell responses, again raising the question whether there are contexts warranting the application of unmodified mRNA vaccines.

M1Ψ has been shown to both attenuate mRNA-induced activation of multiple innate immune receptors and enhance mRNA translation in a wide range of *in vitro* and *in vivo* models.^2^ Its immunomodulatory effect stems from both direct interference with Toll-like receptor (TLR)7 and TLR8 activation and reduced production of immunogenic double-stranded RNA byproducts during *in vitro* transcription. Increased translation is believed to result from both the alleviation of immune-mediated translational restriction and direct, albeit currently unknown, effects on the translational machinery.

The use of m1Ψ is also considered essential to the success of the mRNA-1273 (Moderna) and BNT162b2 (BioNTech) vaccines, which were both over 90% effective at preventing infection,^2^^,^^3^ while the unmodified mRNA vaccination from CVnCoV (CureVac) was only 47% effective^3^ although it used the same lipid nanoparticle (LNP) formulation as in BNT162b2.^4^ A later, optimized version CV2CoV demonstrated similar responses to BNT162b2 in a preclinical NHP model,^4^ but, as of July 2025, clinical data are still pending. Nonetheless, CureVac has now also used m1Ψ in their influenza and SARS-CoV-2 vaccines, including CV0501, CV0601, and CV0701, although head-to-head comparisons have yet to be published.

Beyond antiviral prophylaxis, mRNA therapies have been developed for a variety of other indications, all of which may tolerate (or even require) different degrees of innate immune activation. While, for applications such as protein substitution, minimizing the immune response seems paramount, mRNA-based anti-tumor vaccination, particularly of cold tumors, could conceivably benefit from strong activation of multiple immune receptors to counteract the anti-inflammatory tumor microenvironment. CureVac’s CVGBM cancer vaccine uses unmodified mRNA with promising phase 1 results as a monotherapy (NCT05938387), and BioNTech is also investigating the anti-tumoral potential of non-modified mRNA in its iNest platform, with the lead candidate cevumeran in multiple clinical trials in combination with programmed death-ligand 1 (PD-L1) blockade (NCT03289962, NCT03815058, and NCT05968326).

In their study, the Loré group aimed to model therapeutic cancer vaccines. Although they do not use a tumor antigen in their mRNA construct but rather Gag, a model antigen with known antigenicity, high doses and multiple immunizations were used. The application of 160 μg of unmodified mRNA and 400 (low-dose) or 800 μg (high-dose) of m1Ψ-RNA ranged from 4 to 26.6 times the amount of mRNA used in NHP studies of SARS-CoV-2 vaccination. Moreover, vaccines were applied 5 times every 2 weeks with a sixth booster after 20 weeks (Figure 1). This regimen is roughly in line with the maximal doses in use in clinical trials (NCT03897881, NCT04526899, NCT04534205, and NCT05938387), although doses and regimens vary considerably. Moreover, while identical dosage of modified and unmodified mRNA would conceivably have provided a better direct comparison, the proportions used in the study were chosen to reflect feasible clinical application.

Unmodified mRNA induced higher levels of interferon-alpha (IFNα) and interleukin (IL)-7 than m1Ψ-mRNA although a much lower dose was used. This difference in IFNα induction is not surprising, as unmodified mRNA should activate TLR7 in plasmacytoid dendritic cells (pDC), TLR8 in monocytes, and the RIG-I-like receptors (RLR) more robustly in a variety of cells,^2^^,^^5^ all of which induce type-I IFN release. In contrast, the mechanisms underlying the induction of IL-7 seem less clear. While IL-7 is generally seen as a constitutively expressed cytokine in lymphoid organs, its expression can be induced by TLR activation in the liver,^6^ which could potentially occur after high-dose unmodified mRNA administration, although this direct effect has not yet been shown. Nonetheless, regardless of its source, IL-7 is generally considered T cell supportive and beneficial for the cytotoxic response.

In contrast, m1Ψ-mRNA application resulted in increased IL-6 and tumor necrosis factor (TNF) release. This is unlikely to be the result of RNA modification per se. However, the higher levels of m1Ψ-mRNA utilized also required more LNP, which has been shown to induce TNF and IL-6^7^ on its own and thus crucially support the T follicular helfer (T_fh_) cell and antibody response to m1Ψ-mRNA vaccination. Of note, the same study reported that this effect was independent of Myd88 and, thus, TLR activation, which could conceivably extend to the TLR7/8 activity of unmodified mRNA. Altogether, these higher levels of IL-6 and TNF may be important contributors to the immunogenicity of m1Ψ-mRNA formulations through the concurrently increased levels of LNP used for their delivery.

The authors also observed that unmodified RNA resulted in a tolerizing effect upon repetitive application, with the fifth application of unmodified RNA resulting in less differentially regulated genes (DEGs) than the first and also a reduction in the expression of IFNα, IL-7, and IL-7 downstream targets. Interestingly, DEGs were also reduced for low-dose m1Ψ-mRNA administration, but IL-6 levels remained generally unaltered, and, for high-dose m1Ψ-mRNA, there was no tolerance measurable in any of the readouts. These differences may result from the diverse TLR activity of unmodified mRNA since TLR3, 7, and 8 activation has been reported to induce innate immune tolerance,^8^ while RLR activation, such as what has been observed for BNT162b2,^9^ to date, has not, although this would require a comparative investigation of potential epigenetic changes induced by modified and unmodified mRNA vaccinations, which has not yet been performed.

Nonetheless, the question remains what the net consequence of all these differences is on the key readout of vaccine efficacy: the ensuing adaptive response. Based on the data collected by Engstrand et al., the differences in the antibody and T cell response between these three high-dose regimens are minimal. The Gag-specific antibody titer looks virtually identical, in line with a previous study directly comparing the anti-spike IgG titers induced by CV2CoV (unmodified RNA) and BNT162b2 (m1Ψ RNA).^4^ (Although the sequence of the precise Gag construct used is not in the paper, the methods suggest that the Gag mRNA construct has undergone the same sequence optimization as CV2CoV.) For the T cell compartment, the differences are a bit murkier. Clearly, all three vaccine formulations induce a CD4^+^ and CD8^+^ T cell response, but there do seem to be some differences: a better induction of CD4^+^ memory cells by m1Ψ-mRNA and more IFNγ release from CD8^+^ T cells after unmodified mRNA. However, as is understandable in an NHP study, the sample size remains too small to draw definitive conclusions. Here, one can only hope that further studies in NHPs, but also mice and humans, will continue to expand on these investigations, perhaps with a deeper investigation of the T cell compartment.

Indeed, to date, only two other studies since the COVID pandemic, which we are aware of, have directly compared unmodified and m1Ψ-modified mRNA formulations in NHPs.^4^^,^^10^ There are of course many reasons for this that go beyond the scope of this commentary. However, the current manuscript by Engstrand et al. does address this important gap in the literature, and we hope that more open-ended, head-to-head comparisons of mRNA vaccine formulations will follow, as this seems the most efficient way to optimize therapeutic approaches in an area with an almost inexhaustible clinical need.

## Acknowledgments

This work was supported by the 10.13039/501100001659Deutsche Forschungsgemeinschaft (DFG, German Research Foundation) under Germany’s Excellence Strategy (EXC2151–390873048) (E.B., S.S.) and by 10.13039/501100001659DFG TRR237 (369799452) (E.B., T.Z., K.M.).

## Declaration of interests

The authors declare no competing interests.
